# Humeral Fracture in a Young CrossFit Practitioner

**DOI:** 10.7759/cureus.39781

**Published:** 2023-05-31

**Authors:** Diogo Costa, Rui Brito, Sara Afonso, Nuno Ramalhão, Pedro Cantista

**Affiliations:** 1 Physical Medicine and Rehabilitation, Centro Hospitalar Universitário de Santo António, Porto, PRT

**Keywords:** elbow, distal, humeral fracture, injuries, crossfit

## Abstract

CrossFit (CrossFit Inc, Washington, DC) is a recent, high-intensity strength and conditioning sport that is growing in popularity worldwide. Potential risks and injuries have been described in previous reports. Distal humeral fractures without direct trauma were related to sports like baseball or wrestling. However, they have never been reported in a CrossFit athlete.

We present the first case of distal humeral fracture associated with a CrossFit workout, during a gymnastic movement. Our patient had no relevant medical history but the investigation revealed reduced vitamin D levels and low bone density. The patient was surgically treated and he completed the rehabilitation program. He returned to sports practice 12 weeks after the surgery.

## Introduction

CrossFit (CrossFit Inc, Washington, DC) is a sport that was established in the early 2000s in the United States [1]. The sport has grown in popularity worldwide, with more than 10,000 affiliated gyms [1]. It combines strength and conditioning, gymnastic movements, Olympic weightlifting, powerlifting, and other functional movements into a constantly varied and high-intensity workout [1]. Most of the exercise movements are performed in a limited period with little or no rest [2]. The workouts are scalable to the athlete’s level of physical activity so that the exercises are carried out safely and effectively [3]. It was originally introduced to train individuals whose work required physical fitness and muscle strength (e.g., police officers, military special forces), but it has been quickly adopted by many civilian gymnasiums [2].

Some studies have reported the benefits of this sport such as an increase in maximal aerobic capacity, improvement of body condition, decreased blood pressure, weight loss, and increased insulin sensitivity [1,2,4]. However, there are also risks to this new fitness modality; some musculoskeletal injuries have been reported [2,5] with the shoulders, back, and knees being the most frequently injured body parts [6].

Injury rates in CrossFit are comparable with established injury rates for other recreational or competitive athletes, with an injury profile similar to gymnasts, Olympic weightlifters, and power lifters [2,6]. The rate of injuries is variable (depending on the study), between 0.2 and 18.9 per 1,000 hours of training, and the prevalence of lesions is around 35% [6].

Previous cases have reported severe lesions related to CrossFit such as rhabdomyolysis after exercise [7], traumatic tear of the latissimus dorsi myotendinous junction [8], and even a dissection of the internal carotid artery [9]. Fractures of the upper limbs have also been reported such as a humeral stress fracture [10] and a scapular stress fracture [11].

However, we are not aware of any previously reported case of a distal humeral fracture related to CrossFit in the literature. We therefore describe a clinical case of a fracture at the distal third of the humerus, in a CrossFit practitioner during a gymnastic movement (without direct trauma), to raise awareness about the risk of severe injuries in this new sport modality.

## Case presentation

**Figure 1:**
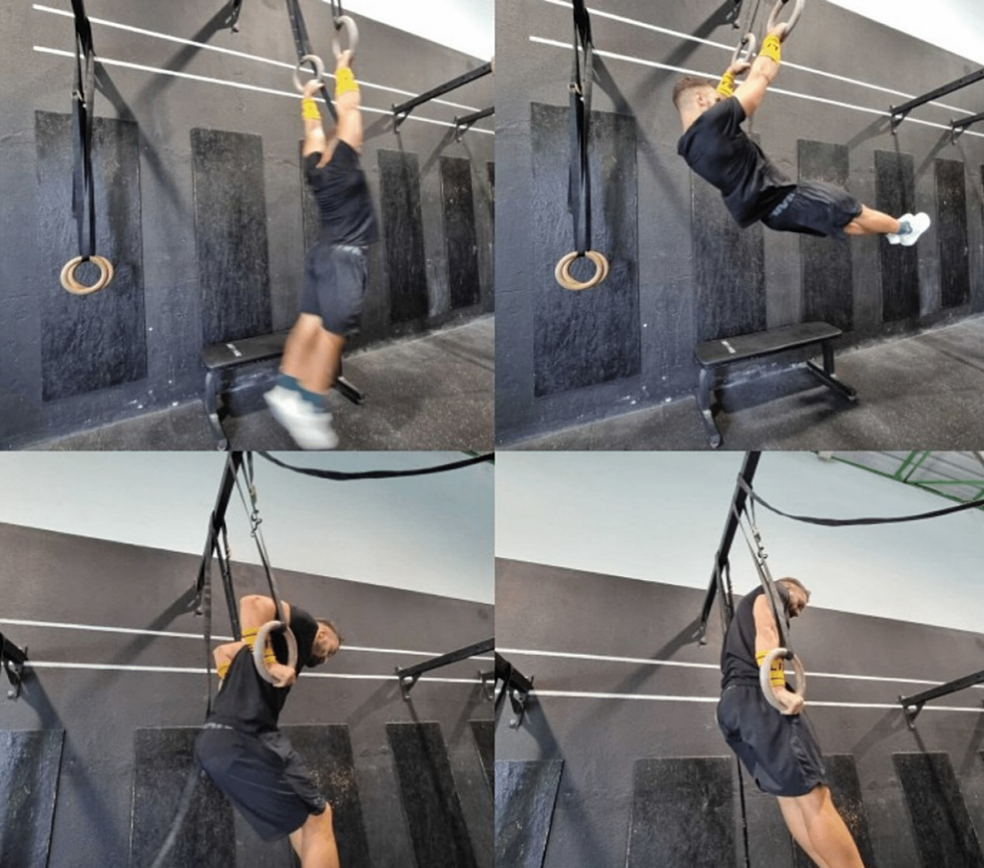
Execution of a ring muscle up

A 38-year-old male presented to the emergency department of our hospital complaining of right arm pain. He was performing a ring muscle up, which is a gymnastic exercise on rings, and heard a loud pop while he was trying to climb onto the rings (Figure 1). He felt immediate severe pain and functional impotence of the right upper limb. The patient denied any trauma or fall. 

A physical examination revealed swelling, ecchymosis, and extreme sensitivity to touch in the right arm. There were no clinical signs of neurovascular injury. His right shoulder and elbow examinations were normal except for a reduced range of motion secondary to pain over his distal arm.

The patient had no relevant medical history, including previous fractures, neoplastic disease, or surgeries on the upper limbs. He denied taking medication such as anabolic or glucocorticoid steroids and did not follow any specific diet, nutritional supplementation, or reduced caloric intake plan. Before starting this modality, the patient had practiced swimming for eight years and had been physically active since he was 16 years old, without any previous relevant injuries in sports training. He started his CrossFit practice three years ago and carried out an average of four workouts per week without engaging in any other sports on the remaining days. The patient denied any changes in volume or intensity from usual training.

**Figure 2:**
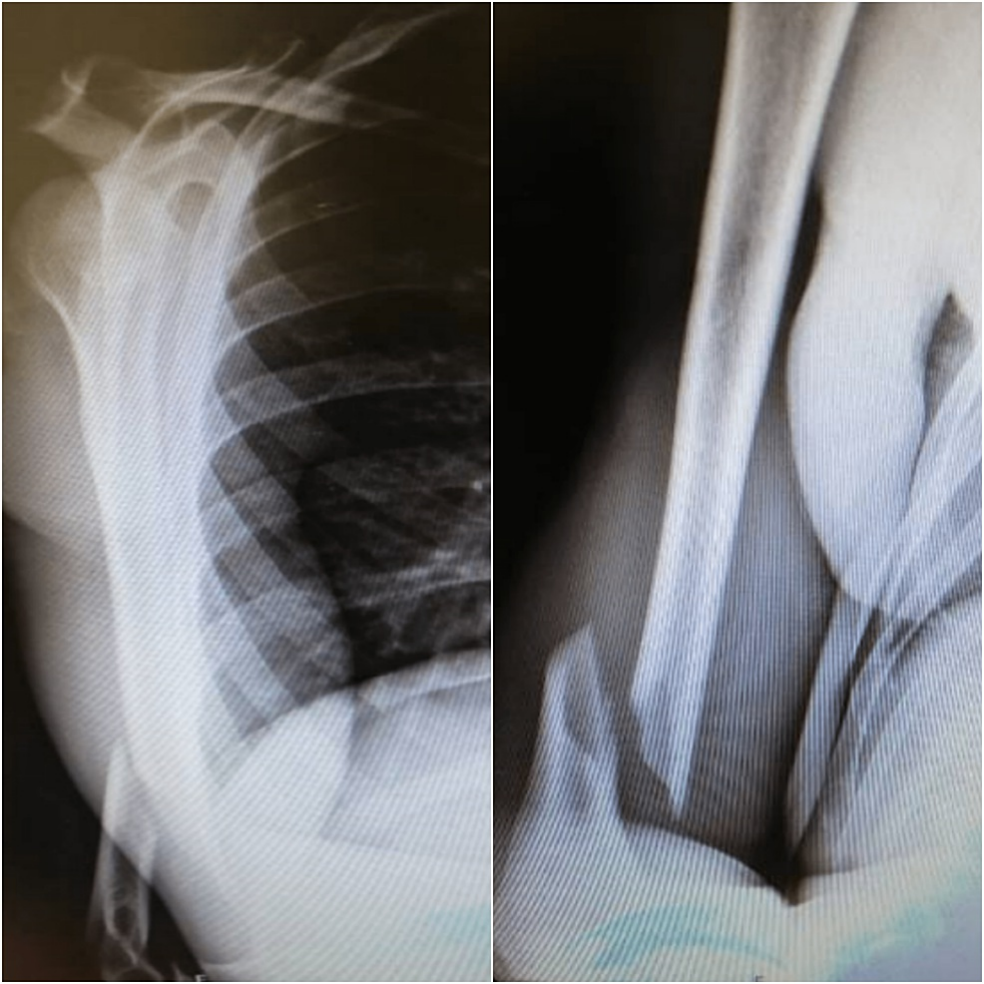
Complete oblique fracture at the distal third shaft of the right humerus Type A fracture - extra-articular (supracondylar fracture) by the AO Foundation/Orthopaedic Trauma Association (AO/OTA) classification

Radiographs were made on the same day and they showed a complete fracture at the distal third shaft of the humerus (Figure 2). There was no radiographic evidence of pathologic bone fracture.

**Figure 3:**
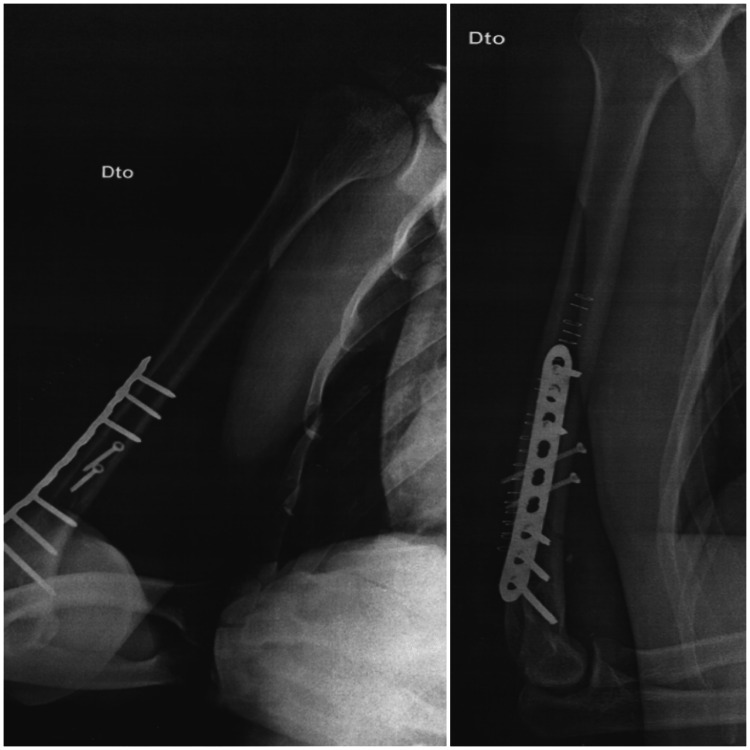
Postoperative view of the humerus with reduction and plate fixation

The patient underwent surgery on the same day, with open reduction and internal fixation with a plate and screws (Figure 3). He was instructed to use a sling for four weeks and started active mobilization exercises for the shoulder, wrist and fingers, and gentle self-passive and active mobilization of the elbow 48 hours after the surgery. To reduce swelling, he was advised to put ice on the arm, four to six times per day. Compression was also recommended. For analgesia, he was prescribed diclofenac 75mg maximum two times per day, and paracetamol 1g for a maximum of three times per day.

He was observed four weeks later in a medical check and presented a limitation of 30 degrees of extension of the elbow. He reached a maximum of 90 degrees of flexion and had a limitation of 45 degrees of pronation and supination of the right elbow during passive mobilisation.

We referred him to an endocrinology consultation for an etiological investigation and he started a rehabilitation program that consisted of a one-hour session of physical therapy every weekday. Range of motion and stretching exercises, especially on the elbow, were progressively performed, and the patient received massage and joint mobilisation. As the patient’s pain decreased, strength and stability exercises were started, first with bands and then with weights. Balance and coordination exercises were conducted such as throwing a ball or catching objects.

**Figure 4:**
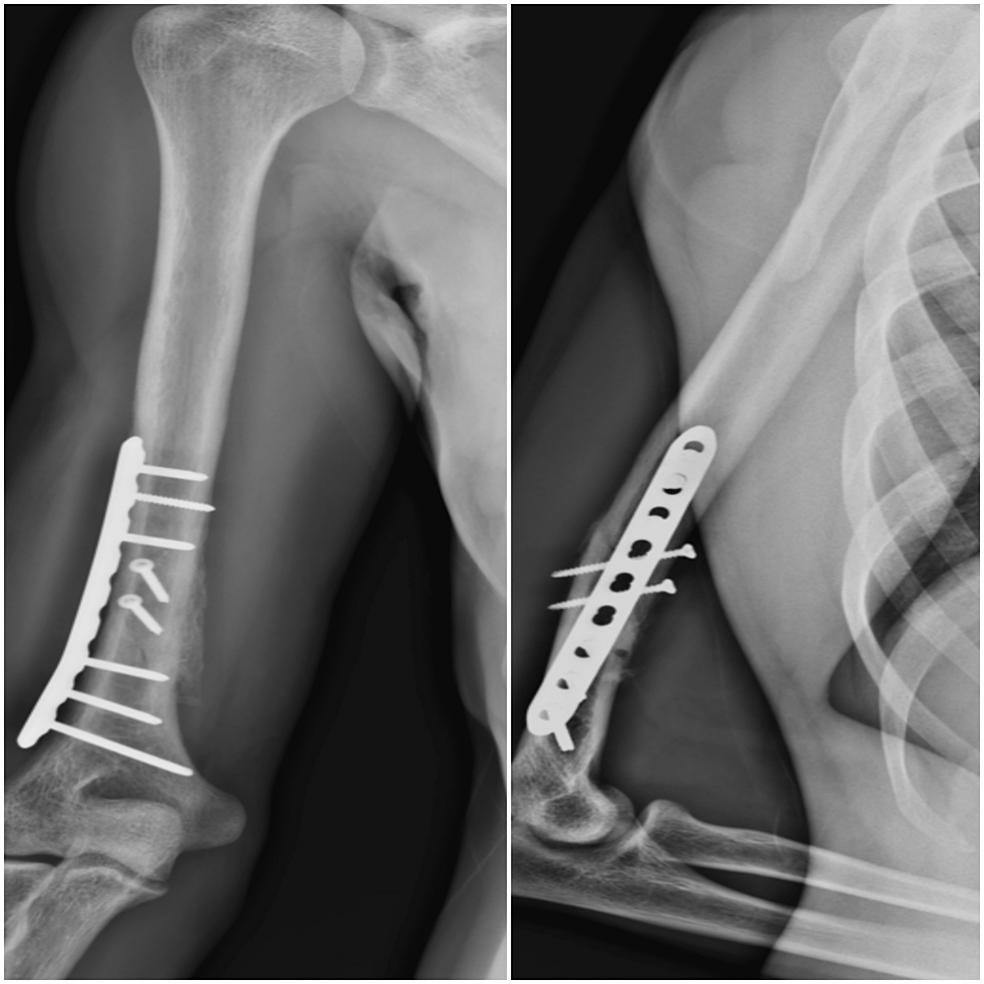
Two months after surgery, already with apparent bone callus formed

**Figure 5:**
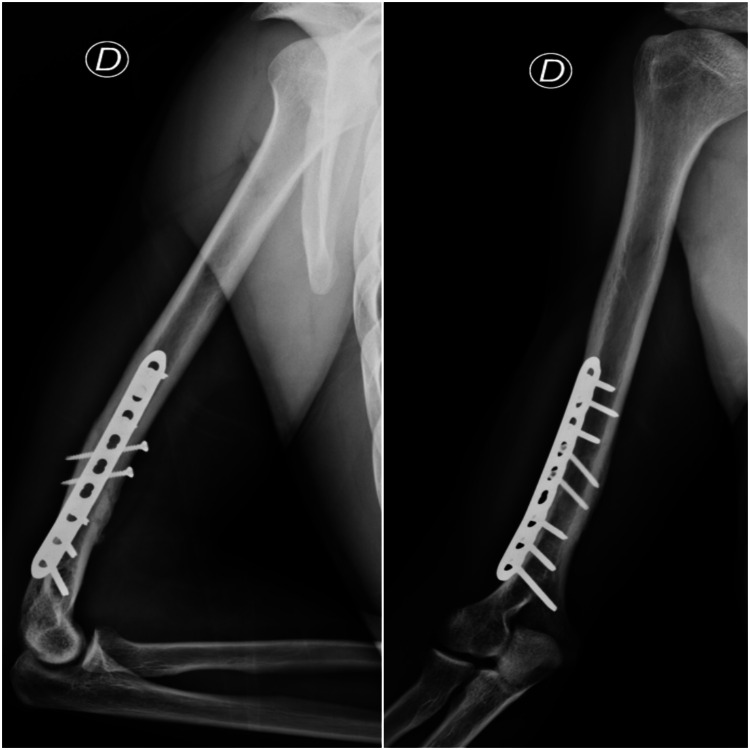
Five months after surgery, with complete bone callus formed

Follow-up X-rays were requested two and five months after surgery which revealed a complete bony callus (Figures 4-5). There was a favourable evolution, and we discharged the patient from rehabilitation 12 weeks after the surgery because he had no deficits in muscle strength or amplitude. He had no pain, scoring 0 on the Numeric Rating Scale (NRS), and had no disability, scoring 0 on the Disability of Arm, Shoulder and Hand (DASH) Questionnaire. After the discharge, the patient then gradually returned to CrossFit training.

Laboratory tests were performed and only revealed Vitamin D insufficiency (serum Vitamin D of 15,9 ng/mL). Calcium, phosphorus, parathyroid hormone, thyroid hormones, urine cortisol, and testosterone serum values were normal. A densitometry was requested, showing a decrease in bone mineral density in the spine, total femur, and femoral neck (Z-SCORE of -0.6, -1.0, and -1.0 respectively) and loss of bone mass relative to a standard deviation in the total femur and femoral neck (T-SCORE of -1.4 and -1.7 respectively), revealing the presence of osteopenia. For these reasons, vitamin D was prescribed (colecalciferol 3335 IU per day) for six months. After the six-month period, the laboratory tests were repeated, revealing no alterations (normal serum Vitamin D value of 38.2 ng/ml), and the patient was discharged from the endocrinology consultation.

Until today, the athlete has been doing his usual training of CrossFit four times per week without any limitations.

## Discussion

Distal humeral fractures have been described in sports such as baseball or arm wrestling as putting excessive stress on the upper limb’s extremities [12,13].

In baseball pitching, the shoulder and the elbow are put in excessive external rotation with consequent elbow valgus [12]. The change from external rotation to internal rotation of the shoulder and elbow extension occurs during the acceleration phase, which is also when the distal humerus experiences the greatest amount of torsional force [12]. Humeral fractures have also occurred with other types of throwing including hand grenades, javelins, and dodge balls [12].

In arm wrestling, the shoulder is flexed at 45º, and the humerus is subjected to forces of internal rotation at the shoulder joint with the actions of the pectoralis major, latissimus dorsi, subscapularis, and teres major [13]. The biceps brachi, brachioradialis, and brachialis are isometrically contracted when the elbow is in fixed flexion [13]. The flexors and pronators of the wrist are isometrically contracted at first with the wrist in a semi-supinated position. The humerus, which is a hollow cylinder, is bent, compressed, and put under torsional strain [13]. Biomechanical studies have shown that the distal third of the humerus is predisposed to injury because of the unfavourable ratio of inner to outer diameter of the bone in this area [13].

In this case, the incident happened in the final transition of the ring muscle ups, with a mechanism similar to arm wrestling. At the time of the fracture, the shoulder was flexed and in the transition to internal rotation, the elbow was flexed and the wrist was in transition from supination to pronation, creating excessive tension in the distal humerus. The patient attempted to transition the movement for at least five seconds with constant isometric contraction and torsion of the humerus which may have led to increased stress and the consequent fracture.

The fracture may have been caused by bone overload from the injury mechanism associated with repetitive and intense CrossFit movements. During the workouts, some movements are performed at high repetition range, at high intensity and with heavy weights and complex gymnastic movements which may lead to poor form and placing the upper limb at extremes of motion in a position that increases the likelihood of an injury [14]. High-intensity interval training, repetitive overhead weightlifting, powerlifting, kettlebell movements, handstand push-ups and gymnastic movements such as muscle-ups cause both repetitive muscle action on the humerus and muscle fatigue [14]. Practitioners of this modality go through numerous intense training sessions, during which the muscles being trained often hypertrophy faster than the bone can remodel [11].

Other risk factors that seem to have been determinants for the occurrence of this injury associated with intense CrossFit training were osteopenia and vitamin D (25(OH) D) deficiency. At the time of the fracture, the patient had low levels of vitamin D and a densitometry revealed low bone density.

Athletes with low bone density (osteopenia) may be at an increased risk of fractures compared to those with normal bone density. Maintenance of bone mass can reduce fracture risk by 50% to 80% [15]. Vitamin D is crucial for bone metabolism and overall bone health in active people [16]. Studies report that vitamin D deficiency can lead to stress fractures in athletes, and there is a relationship between low levels of vitamin D and an increased risk of stress or insufficiency fractures [16]. The intake of 25(OH)D is often insufficient for the training volume of an athlete because of inadequate recovery, increased bone turnover from repetitive stress, and deficiencies in dietary intake [16]. For some athletes, getting enough vitamin D seems to be beneficial, especially in sports where body weight is a key factor in performance outcomes [16]. The patient achieved normal serum levels of vitamin D after six months of supplementation (38.2 ng/ml).

## Conclusions

In this report, a young athlete experienced an indirect trauma distal humeral fracture caused by mechanical overload during CrossFit recreational practice. CrossFit is a modality that includes exercises with high loads and a high number of repetitions that condition muscle fatigue and microtrauma phenomena. These two phenomena seem to have had a revealing role in the pathophysiology of this case. The presence of twisting trauma in this clinical case and the young age of the patient motivated the investigation of other etiological factors that contributed to the fracture. The practice of high-intensity physical activity associated with low levels of vitamin D and osteopenia with alteration of the trabecular bone architecture led to humeral fracture, which did not cause sequelae phenomena in this case.

As CrossFit continues to gain popularity all over the world, clinicians should be aware of the risks of this sport. Our case report is the first description of a distal humeral fracture in this sport, and additional investigational studies to identify the incidence of CrossFit-related injuries and their characterisations are necessary.
